# Vitamin D status and site-specific fracture pattern associations in older adults with fragility fractures: a cross-sectional analysis of 2543 patients

**DOI:** 10.3389/fnut.2026.1810545

**Published:** 2026-04-24

**Authors:** Weifeng Lv, Junwei Lin, Kun Lin, Xiaoqing Wen, Qi Lin, Yao Li, Jinxiang Zeng, Qianying Nie, Mingqiang Guan, Rong Su

**Affiliations:** 1 Department of Laboratory Medicine, Foshan Hospital of Traditional Chinese Medicine, Guangzhou University of Chinese Medicine, Foshan, Guangdong, China; 2 The Eighth Clinical Medical College of Guangzhou University of Chinese Medicine, Foshan, Guangdong, China; 3 Foshan Key Laboratory of Chinese Medicine, Foshan Hospital of Traditional Chinese Medicine, Guangzhou University of Chinese Medicine, Foshan, Guangdong, China; 4 Department of Orthopedics, Foshan Hospital of Traditional Chinese Medicine, Guangzhou University of Chinese Medicine, Foshan, Guangdong, China; 5 Guangdong Provincial Clinical Research Center for Laboratory Medicine, Guangzhou, Guangdong, China

**Keywords:** cross-sectional analysis, fracture site, fragility fracture, older adults, vitamin D deficiency

## Abstract

**Background:**

Vitamin D deficiency is an established risk factor for osteoporosis and fragility fractures in the elderly. However, its prevalence and association with specific anatomic fracture sites remain incompletely understood. This study aimed to assess vitamin D status and investigate its variation across fracture sites in elderly patients with fragility fractures.

**Methods:**

A total of 2543 hospitalized patients aged ≥60 years with fragility fractures were included. Serum 25(OH)D concentrations were measured by liquid chromatography–tandem mass spectrometry. Demographic characteristics, admission indications, and comorbidities were extracted from electronic medical records to assess the prevalence of vitamin D deficiency and identify associated risk factors. A generalized linear model with a gamma distribution was used to analyze the association between serum 25(OH)D concentration and fracture site, adjusting for age, sex, sampling season, and comorbidities.

**Results:**

Overall, 35.9% of patients were vitamin D deficient (<20 ng/mL), 44.2% were insufficient (20–30 ng/mL), and 19.9% were sufficient. The median serum 25(OH)D concentration was 22.60 ng/mL (IQR: 17.30–28.30). Multivariable analysis showed that age (OR 1.036 per year), female sex (OR 1.358), winter season (OR 2.040), and a history of cerebral infarction (OR 1.660) were independently associated with vitamin D deficiency (all *p* < 0.05). Serum 25(OH)D concentrations differed significantly across fracture sites (*p* < 0.05): hip fractures (22.20 ng/mL) and multiple fractures (20.95 ng/mL) had the lowest levels, while vertebral fractures (25.40 ng/mL) had the highest.

**Conclusion:**

Vitamin D deficiency is highly prevalent in older adults with fragility fractures and is significantly associated with advancing age, female sex, winter season, and a history of cerebral infarction. After multivariable adjustment, serum 25(OH)D concentrations differed significantly across fracture sites. This difference may reflect the dual role of vitamin D in neuromuscular function and bone metabolism, underscoring the need for longitudinal interventional studies to further explore these relationships.

## 1 Introduction

Osteoporosis is a systemic skeletal disorder characterized by decreased bone mass and progressive deterioration of bone microarchitecture, representing a significant public health concern worldwide (1). The number of patients with osteoporosis is expected to increase with rising life expectancy, lifestyle changes, and global population aging (2, 3). Osteoporosis-related fragility fractures typically result from low-energy trauma and represent a serious complication of the disease (4, 5). It not only significantly impairs patients’ physical and mental health but also imposes a considerable economic burden on families and society. The most common types of fragility fractures include hip, wrist, and vertebral fractures, with the elderly and postmenopausal women representing the primary high-risk populations (6). Studies have reported that the worldwide lifetime risk of osteoporotic fragility fractures is as high as 40% in women and about 13% in men; moreover, 11% of patients may experience secondary fractures within 3 years (6, 7). In addition to a high incidence, the disability and mortality rates associated with fragility fractures have remained persistently high. Studies have reported that the 1-year mortality rate among elderly patients after hip fracture is as high as 17–33%, and the overall mortality rate is estimated to be approximately 20% (8–10). Therefore, the treatment of osteoporosis and research into related risk factors are of significant importance in mitigating the substantial social and economic burden associated with osteoporosis-related fragility fractures.

Vitamin D is a crucial nutrient that plays a significant role in maintaining bone health and structural integrity (11). Although vitamin D can be obtained through sunlight exposure, dietary sources, and supplementation, deficiency remains a global health concern. It is estimated that approximately one billion people worldwide have vitamin D deficiency, and 50% of the global population have vitamin D insufficiency (12, 13). Numerous studies have shown that vitamin D deficiency or insufficiency can cause impaired bone mineralization, secondary hyperparathyroidism, and progressive decline in cortical bone mineral density. These pathophysiological alterations collectively constitute an important mechanism for the development and progression of osteoporosis and its related fragility fractures (14–16). For example, a case–control study showed that low serum 25(OH)D concentrations are associated with an increased risk of hip fracture in older adults (15). Another study demonstrated that vitamin D insufficiency is an independent risk factor for vertebral fragility fractures in both men and women (16). In addition, a multicenter prospective study showed that lower levels of serum vitamin D were significantly associated with unstable intertrochanteric fractures (17). However, existing studies largely concentrate on the association between vitamin D status and the risk of fragility fractures at particular anatomical locations. Evidence is still lacking to support a comprehensive understanding of the vitamin D nutritional profile across the entire fragility fracture cohort, as well as systematic comparisons of serum 25(OH)D concentrations between patients with fractures at distinct anatomical sites. In clinical practice, fragility fractures predominantly occur at sites such as the hip, wrist, and vertebrae, which exhibit substantial disparities in etiology, biomechanical properties, and clinical outcomes. Whether vitamin D deficiency is differentially associated with these distinct fracture types remains unclear. Therefore, this study aims to comprehensively characterize the vitamin D nutritional status in patients with fragility fractures and, through a systematic comparison of serum 25(OH)D concentrations among patients with fractures at different anatomical sites, clarify the association between vitamin D deficiency and fracture type.

## 2 Methods

### 2.1 Study design

This study is a single-center, cross-sectional, observational study conducted at Foshan Hospital of Traditional Chinese Medicine (23.04° N, Foshan City, Guangdong Province, China). It included elderly patients aged ≥60 years who were hospitalized for fragility fractures between September 2023 and August 2025. The fragility fractures referred to in this study are hip, wrist, and vertebral fractures resulting from low-energy trauma (e.g., a fall from standing height or lower, or a simple fall on level ground). Data from patients who met all of the following inclusion criteria were used for analysis in this study: (1) age ≥60 years at enrollment; (2) a fragility fracture resulting from low-energy trauma; (3)available serum 25(OH)D concentration measurement. Exclusion criteria for this study were: (1) traumatic fracture caused by high-energy trauma (e.g., motor vehicle accident); (2) pathological fracture secondary to malignancy, multiple myeloma, or metabolic bone disease; (3) non-recent fractures. The study was conducted in accordance with the Declaration of Helsinki and approved by the Ethics Committee of Foshan Hospital of Traditional Chinese Medicine.

### 2.2 Data collection and definition

All data were extracted from electronic medical records and included age, sex, admission date, fracture location, primary reason for admission, and comorbidities. Serum 25(OH)D concentrations in all patients were measured using liquid chromatography–tandem mass spectrometry (LC–MS/MS). For data evaluation, vitamin D nutritional status was categorized as deficiency [25(OH)D < 20 ng/mL], insufficiency [20 ng/mL ≤ 25(OH)D < 30 ng/mL], or sufficiency [25(OH)D ≥ 30 ng/mL] (18). It should be noted that there is currently no international consensus on the criteria for defining vitamin D deficiency, and the serum 25(OH)D thresholds proposed by different authoritative guidelines vary. Considering that approximately 90% of human vitamin D is synthesized cutaneously in a UVB-dependent manner, this study used the patient′s admission month as a proxy variable for seasonal sun exposure. Based on the long-term climatological characteristics of Foshan City, patients were categorized into four groups: spring (March–May), summer (June–September), autumn (October–November), and winter (December–February). In this region, September maintains sunshine duration and climatic conditions comparable to the summer months, as documented by local meteorological records, supporting the inclusion of September within the summer season.

### 2.3 Statistical analysis

All statistical analyses were performed using IBM SPSS Statistics software (Version 27). In the analysis of patients’ characteristics, continuous variables were reported as mean (standard deviation, SD) or median (interquartile range, IQR), depending on their distribution; categorical variables were presented as frequencies and percentages. Differences among normally distributed continuous variables were assessed using one-way analysis of variance (ANOVA). The Kruskal–Wallis test was applied for multiple-group comparisons of non-normally distributed continuous variables. Categorical variables were compared using the chi-square test. Multivariable binary logistic regression was used to identify risk factors for vitamin D deficiency (defined as serum 25(OH)D < 20 ng/mL), with results reported as odds ratios (ORs) with 95% confidence intervals (CIs). Reference categories were defined as male (for sex) and summer (for sampling season). Given that serum 25(OH)D concentrations are strictly positive, continuous, and typically exhibit a right-skewed distribution (Figures S1 and S2), a generalized linear model (GLM) with a gamma distribution and log link was employed to examine the association between serum 25(OH)D concentrations and fracture site. The model was adjusted for age (continuous), sex (reference: male), sampling season (reference: summer), and comorbidities. Fracture site was entered as a categorical variable with vertebral fractures as the reference category. Model results are presented as coefficient estimates (*β*), exponentiated coefficients [Exp(β)] representing the multiplicative change in the geometric mean of serum 25(OH)D, with corresponding 95% confidence intervals and *p*-values. For multiple comparisons, the Bonferroni correction was applied. *p* values < 0.05 were considered statistically significant.

## 3 Results

### 3.1 Baseline characteristics of study patients according to vitamin D nutritional status

A total of 2,543 fragility fracture patients were evaluated in this study, with 914 (35.94%) classified as vitamin D deficient, 1,123 (44.16%) as insufficient, and 506 (19.90%) as sufficient. As summarized in Table 1, significant differences were observed across these groups. Patients with vitamin D deficiency were significantly older and more likely to be female. A strong seasonal variation was evident, with vitamin D deficiency being most prevalent in winter (*p* < 0.001). Regarding comorbidities, a history of cerebral infarction and coronary artery disease was significantly associated with a higher prevalence of vitamin D deficiency. No significant associations were observed between vitamin D status and other comorbidities, including malignancy, chronic obstructive pulmonary disease (COPD), chronic kidney disease, hypertension, hepatobiliary disease, and diabetes.

**Table 1:** Characteristics of the study participants according to vitamin D nutritional status.

Characteristics	Total	Vit-D sufficiency	Vit-D insufficiency	Vit-D deficiency	*p*-value
No. of patients	2543	506	1123	914	
Age (years)	76.82 (±8.99)	75.05 (±9.10)	75.95 (±8.85)	78.86 (±8.70)	< 0.001*
Sex					< 0.001*
Male	736 (28.94%)	176 (34.78%)	332 (29.56%)	228 (24.95%)	
Female	1807 (71.06%)	330 (65.22%)	791 (70.44%)	686 (75.05%)	
Sampling season					< 0.001*
Spring	614 (24.14%)	111 (21.94%)	271 (24.13%)	232 (25.38%)	
Summer	608 (23.91%)	159 (31.42%)	293 (26.09%)	156 (17.07%)	
Autumn	520 (20.45%)	90 (17.79%)	251 (22.35%)	179 (19.58%)	
Winter	801 (31.50%)	146 (28.85%)	308 (27.43%)	347 (37.96%)	
Comorbidities					
History of malignancy	65 (2.56%)	15 (2.96%)	27 (2.4%)	23 (2.52%)	0.799
Diabetes	507 (19.94%)	93 (18.38%)	227 (20.12%)	187 (20.46%)	0.613
COPD	48 (1.89%)	10 (1.98%)	19 (1.69%)	19 (2.08%)	0.805
History of cerebral infarction	306 (12.03%)	38 (7.51%)	124 (11.04%)	144 (15.75%)	< 0.001*
Chronic kidney disease	85 (3.34%)	12 (2.37%)	36 (3.21%)	37 (4.05%)	0.229
Hypertension	1,227 (48.25%)	250 (49.41%)	518 (46.13%)	459 (50.22%)	0.156
Coronary artery disease	248 (9.75%)	49 (9.68%)	89 (7.93%)	110 (12.04%)	0.008*

Data are presented as mean ± standard deviation or number (percentage). COPD, chronic obstructive pulmonary disease; *variables with *p*-value < 0.05.

### 3.2 Analysis of serum 25(OH)D concentrations across different demographic groups

The median serum 25(OH)D concentration of the entire patient population was 22.60 ng/mL (IQR: 17.30–28.30), with 23.60 ng/mL (IQR: 18.30–29.70) for males and 22.20 ng/mL (IQR: 17.00–27.80) for females (Table 2). No cases of vitamin D toxicity (25(OH)D > 100 ng/mL) were identified. The serum 25(OH)D concentrations were significantly higher in male patients than in female patients (*p* < 0.001). Significant differences were also observed across different age and seasonal groups. A clear trend was observed across age groups, with serum 25(OH)D concentrations decreasing with increasing age; this decline was statistically significant between groups with larger age spans (Supplementary Table S1). Furthermore, serum 25(OH)D concentrations demonstrated significant seasonal variation (*p* < 0.001). The lowest levels were observed in winter: 21.60 ng/mL (IQR: 16.50–27.50), while the highest levels were recorded in summer: 24.40 ng/mL (IQR: 19.83–30.58). Serum vitamin D levels were significantly higher in summer compared with the other three seasons (Supplementary Table S2).

**Table 2:** The distribution of 25(OH)D concentrations (ng/mL) across different demographic groups.

Characteristics	*N*	P25	P50	P75	*p*-value
Total	2,543	17.30	22.60	28.30	
Sex					<0.001*
Male	736	18.30	23.60	29.70	
Female	1807	17.00	22.20	27.80	
Age					<0.001*
60-64y	253	20.50	24.20	31.50	
65-69y	364	19.73	24.80	29.80	
70-74y	456	18.05	23.60	28.70	
75-79y	427	17.70	22.60	28.80	
80-84y	452	16.30	21.30	27.18	
≥85y	591	15.30	21.00	26.30	
Season					<0.001*
Spring	614	16.60	22.40	27.90	
Summer	608	19.83	24.40	30.58	
Autumn	520	17.80	22.50	27.68	
Winter	801	16.50	21.60	27.50	

*variables with *p*-value < 0.05.

### 3.3 Factors associated with vitamin D deficiency

To identify the factors associated with vitamin D deficiency, a multivariable logistic regression analysis was conducted. The analysis demonstrated that age, sex, sampling season, and a history of cerebral infarction were associated with an increased risk of vitamin D deficiency (Table 3). With each additional year of age, the odds of vitamin D deficiency increased by 3.6% (OR = 1.036, 95% CI: 1.026–1.046, *p* < 0.001). Female patients had a statistically significant 35.8% higher odds of vitamin D deficiency compared with male patients (OR = 1.358; 95% CI: 1.124–1.641, *p* = 0.001). A robust seasonal effect was observed. Relative to summer, the odds of vitamin D deficiency were significantly elevated in autumn (OR = 1.497, 95% CI: 1.153–1.943, *p* = 0.002), spring (OR = 1.748, 95% CI: 1.363–2.242, *p* < 0.001), and winter (OR = 2.040, 95% CI: 1.614–2.577, *p* < 0.001), with the strongest effect observed during winter. Regarding comorbidities, a history of cerebral infarction emerged as a significant predictor of vitamin D deficiency (OR = 1.660, 95% CI: 1.295–2.129, *p* < 0.001). Coronary artery disease showed a trend toward association but was not statistically significant (*p* = 0.075).

**Table 3:** Logistic regression: predictors of vitamin D deficiency (< 20 ng/mL).

Predictor	OR	95% CI	*p*-value
Age	1.036	1.026–1.046	< 0.001*
Sex [Female]	1.358	1.124–1.641	0.001*
Sampling season [Autumn]	1.497	1.153–1.943	0.002*
Sampling season [Spring]	1.748	1.363–2.242	< 0.001*
Sampling season [Winter]	2.040	1.614–2.577	< 0.001*
History of cerebral infarction	1.660	1.295–2.129	< 0.001*
Coronary disease	1.283	0.975–1.688	0.075

Reference categories: male (for sex), summer (for sampling season). *Variables with *p*-value < 0.05.

### 3.4 Baseline characteristics and serum 25(OH)D concentrations in patients across different fracture sites

As presented in Table 4, among the 2,543 patients with fragility fractures enrolled in the study, 1846 were diagnosed with hip fractures, 105 with wrist fractures, 423 with vertebral fractures, and 169 with multiple-site fractures. Statistically significant differences in age, sex, and seasonal distribution were observed among groups with different fracture sites (all *p* < 0.001). The median serum 25(OH)D concentration also varied significantly by fracture site (*p* < 0.001): vertebral fractures exhibited the highest median level (25.40 ng/mL; IQR: 20.00–37.44), followed by wrist fractures (23.30 ng/mL; IQR: 18.35–35.90), hip fractures (22.20 ng/mL; IQR: 16.90–33.70), and multiple-site fractures (20.95 ng/mL; IQR: 16.18–31.42). The distribution of vitamin D nutritional status also differed significantly across fracture sites (*p* < 0.001). Hip fractures and multiple-site fractures exhibited the highest prevalence of vitamin D deficiency (38.03 and 44.38%, respectively), whereas vertebral fractures showed the highest prevalence of vitamin D sufficiency (28.84%). The complete distribution is reported in Table 4.

**Table 4:** Baseline characteristics and serum 25(OH)D concentrations in patients across different fracture sites.

Characteristics	Hip	Wrist	Vertebral	Multi-site	*p*-value
*N*	1846	105	423	169	
Age (years)	77.83 ± 8.86	70.97 ± 8.15	72.99 ± 9.29	78.94 ± 5.53	< 0.001*
Sex					< 0.001*
Male	602 (32.61%)	14 (13.33%)	90 (21.28%)	30 (17.75%)	
Female	1,244 (67.39%)	91 (86.67%)	333 (78.72%)	139 (82.25%)	
Sampling season					< 0.001*
Spring	431 (23.35%)	30 (28.57%)	104 (24.59%)	50 (29.59%)	
Summer	398 (21.56%)	43 (40.95%)	137 (32.39%)	29 (17.16%)	
Autumn	410 (22.21%)	16 (15.24%)	66 (15.60%)	28 (16.57%)	
Winter	607 (32.88%)	16 (15.24%)	116 (27.42%)	62 (36.69%)	
25 (OH)D concentrations	22.20 (16.90, 33.70)	23.30 (18.35, 35.90)	25.40 (20.00, 37.44)	20.95 (16.18, 31.42)	< 0.001*
Vitamin D nutritional status					< 0.001*
Vit-D deficiency	702 (38.03%)	34 (32.38%)	103 (24.35%)	75 (44.38%)	
Vit-D insufficiency	810 (43.88%)	46 (43.81%)	198 (46.81%)	69 (40.83%)	
Vit-D sufficiency	334 (18.09%)	25 (23.81%)	122 (28.84%)	25 (14.79%)	

Data are presented as mean ± standard deviation or number (percentage); *Variables with *p*-value < 0.05.

### 3.5 Multivariable analysis of factors associated with serum 25(OH)D concentrations

The results of the generalized linear model (gamma distribution, log link) are presented in Table 5. After multivariable adjustment for age, sex, sampling season, and comorbidities, serum 25(OH)D concentrations differed significantly across fracture sites. Relative to patients with vertebral fractures (reference group), those with hip fractures [Exp(*β*) = 0.911; 95% CI: 0.875–0.948; *p* < 0.001] and multiple-site fractures [Exp(*β*) = 0.881; 95% CI: 0.824–0.942; *p* < 0.001] had significantly lower serum 25(OH)D concentrations. Wrist fractures were not associated with a statistically significant difference in serum 25(OH)D concentration [Exp(*β*) = 0.925; 95% CI: 0.855–1.002; *p* = 0.07]. Additional independent predictors of lower serum 25(OH)D concentration included older age [Exp(*β*) = 0.994 per year; 95% CI: 0.993–0.996; *p* < 0.001], female sex [Exp(β) = 0.945; 95% CI: 0.915–0.976; *p* < 0.001], non-summer seasons [autumn: Exp(β) = 0.923; winter: Exp(β) = 0.900; spring: Exp(β) = 0.903; all *p* < 0.001], and a history of cerebral infarction [Exp(β) = 0.909; 95% CI: 0.869–0.951; *p* < 0.001]. *Post-hoc* pairwise comparisons (Supplementary Table S3) confirmed that vertebral fractures had significantly higher 25(OH)D levels than hip and multiple-site fractures (both *p* < 0.001), with multiple-site fractures showing the lowest levels.

**Table 5:** Factors associated with serum 25(OH)D concentrations: results from gamma regression with a log link.

Predictor	Coefficient	Std. error	Exp (B)	95% CI for Exp (B)	*p*-value
Age	−0.006	0.001	0.994	0.993–0.996	<0.001*
Sex	−0.057	0.017	0.945	0.915–0.976	<0.001*
Sampling season					
Autumn	−0.080	0.022	0.923	0.883–0.965	<0.001*
Winter	−0.105	0.020	0.900	0.865–0.936	<0.001*
Spring	−0.102	0.021	0.903	0.866–0.942	<0.001*
Fracture site					
Hip	−0.093	0.021	0.911	0.875–0.948	<0.001*
Wrist	−0.078	0.041	0.925	0.855–1.002	0.07
Multi-site	−0.127	0.034	0.881	0.824–0.942	<0.001*
History of cerebral infarction	−0.095	0.023	0.909	0.869–0.951	<0.001*

Reference categories: male (for sex), summer (for sampling season), vertebral fractures (for fracture site). *Variables with *p*-value < 0.05.

## 4 Discussion

The primary finding of this study is a relatively high prevalence of vitamin D deficiency in the fragility fracture population, and this deficiency was significantly associated with advanced age, female sex, the winter season, and a history of cerebral infarction. Furthermore, we observed statistically significant differences in serum 25(OH)D concentrations among patients with fragility fractures at different anatomical sites after adjusting for confounding factors.

In recent years, multiple studies have demonstrated a high prevalence of vitamin D deficiency among elderly patients with fragility fractures. However, its prevalence exhibits significant heterogeneity across different ethnic groups and geographical regions. For instance, a German cross-sectional study of patients with vertebral fragility fractures—using <12 ng/mL as the cutoff for vitamin D deficiency—reported a deficiency prevalence of 78% (16). A cross-sectional study of Japanese patients with hip fragility fractures reported that, using serum 25-hydroxyvitamin D < 30 ng/mL as the threshold for insufficiency and <20 ng/mL for deficiency, the prevalence of vitamin D insufficiency and deficiency was as high as 93.9 and 71.7%, respectively (19). Similarly, a study of Asian Indian patients with fragility hip fractures reported a vitamin D deficiency (<20 ng/mL) prevalence of 96.7% (20). In contrast, Wang et al.’s cross-sectional study of elderly Chinese patients with osteoporotic hip fractures identified lower rates: 16.67% for deficiency and 66.67% for insufficiency (21). Notably, the present study reports an intermediate prevalence—35.94% for deficiency and 44.16% for insufficiency—among elderly patients with fragility fractures in China. To our knowledge, this is the first large-scale cross-sectional study in China specifically targeting elderly patients with fragility fractures, with the aim of comprehensively assessing vitamin D nutritional status and systematically revealing the epidemiological distribution characteristics of vitamin D deficiency and insufficiency in this population.

Vitamin D deficiency is influenced by multiple factors (22–26). In this study, multivariable analysis of risk factors for vitamin D deficiency among elderly patients with fragility fractures revealed a significant positive association between increasing age and higher deficiency prevalence—a trend consistent with the well-established epidemiological pattern of age-related decline in serum 25(OH)D concentrations, as documented in healthy older adults (22, 23). The gender differences in vitamin D levels remain controversial. This study found that female patients had significantly lower serum 25(OH)D concentrations and a higher prevalence of vitamin D deficiency than their male counterparts—a finding consistent with several cohort studies conducted in Asian populations (14, 22, 24). In contrast, comparable studies from Germany (25) and Austria (26) reported no statistically significant sex-based differences in serum 25(OH)D concentrations. Collectively, these discrepancies suggest that observed sex disparities in vitamin D status are more likely driven by modifiable lifestyle factors—including dietary intake, cultural norms regarding sun exposure, and differential use of sun protection—rather than immutable biological determinants. Such distinctions carry direct clinical implications for tailoring evidence-based vitamin D supplementation and deficiency management strategies. Furthermore, seasonal variation in ultraviolet B (UVB) radiation intensity exerts a robust influence on serum 25(OH)D distribution. Serum 25(OH)D concentrations peaked in summer—coinciding with maximal UVB availability—and reached their annual nadir in winter, when UVB exposure is markedly reduced. Consequently, deficiency prevalence was lowest in summer and highest in winter across both sexes. This inverse relationship underscores UVB radiation as the primary physiological driver of cutaneous vitamin D synthesis. Consistent with our results, numerous studies have reported parallel seasonal patterns, with peak 25(OH)D concentrations in summer and troughs in late winter or early spring (22, 24, 25).

Current research demonstrates that vitamin D deficiency is associated with chronic diseases (24, 26). Therefore, this study focused on examining the impact of several common chronic conditions—including hypertension, coronary heart disease, diabetes, chronic obstructive pulmonary disease (COPD), and tumors—on serum 25(OH)D concentrations in patients with fragility fractures. The results showed that a history of cerebral infarction significantly increased the risk of vitamin D deficiency in patients. In contrast, malignancies, diabetes, COPD, chronic kidney disease, hypertension, and hepatobiliary diseases were not significantly associated with vitamin D deficiency in patients. Consistent with our findings, a cohort study conducted by the Zahir team on hip fracture risk in the elderly also found a significant positive correlation between physical mobility and serum 25(OH)D concentrations (24). It is noteworthy that patients with a prior history of cerebral infarction commonly exhibit reduced mobility, and this condition is closely associated with a significantly increased risk of vitamin D deficiency. The underlying mechanisms likely involve diminished outdoor sun exposure and reduced levels of physical activity—both of which are critical environmental and behavioral factors essential for endogenous vitamin D synthesis (27). Our study also found that coronary heart disease was associated with vitamin D deficiency in univariate analysis (*p* = 0.075), though this association did not reach statistical significance after multivariable adjustment; in contrast, a meta-analysis by Wang et al. reported a significant association between low vitamin D status and elevated cardiovascular disease (CVD) risk (28).

The present study also observed significant differences in serum 25(OH)D concentrations across patients with fragility fractures at distinct anatomical sites. Specifically, patients with hip fractures and those with multiple fractures exhibited the lowest serum 25(OH)D levels, followed by patients with wrist fractures; in contrast, patients with vertebral fractures had the highest concentrations. Existing studies have shown that vitamin D maintains bone strength by promoting intestinal calcium absorption and regulating bone mineralization (29–32). However, if inferred solely from this skeletal-specific action, vitamin D deficiency would be expected to uniformly increase fracture risk across all sites—a pattern that contrasts sharply with the pronounced site-specific distribution observed in our study. Notably, vitamin D also exerts well-established neuromuscular effects: it reduces fall risk and improves postural control during a fall by optimizing muscle fiber composition, enhancing muscle contractile strength, and improving neuromuscular coordination (33, 34). Based on these dual skeletal and muscular functions, we propose a speculative yet physiologically plausible mechanism: vitamin D deficiency may differentially affect bone metabolism and neuromuscular function, thereby influencing fracture susceptibility across anatomical sites.

It should be emphasized that the following mechanistic inferences are reasonable hypotheses based on the cross-sectional observational data from our study and are intended to provide a theoretical framework for future causal research; they are not supported by prospective or interventional evidence. Specifically, the relatively low serum 25(OH)D concentrations and the winter peak in fracture incidence observed in patients with hip fractures suggest the possibility that the pathogenesis of this fracture type may be more closely related to neuromuscular impairment resulting from vitamin D deficiency, which can attenuate protective responses during a fall (e.g., upper extremity bracing reflexes) and thereby increase the risk of fracture from direct force impact to the hip. Patients with vertebral fractures have the highest serum 25(OH)D concentrations, and incidence peaks in summer and autumn. This pattern indicates that the pathogenesis is more likely driven by primary deterioration of bone microarchitecture and progressive loss of bone strength, whereas external factors like falls and vitamin D deficiency contribute relatively less to the disease process. Patients with wrist fractures had the youngest age (70.97 ± 8.15 years) among the four groups, exhibited relatively higher serum vitamin D levels, and showed the highest incidence in summer. These characteristic are consistent with a higher level of daily physical activity and is more likely to initiate a rapid, coordinated protective hand response during a fall; this phenomenon may be closely related to superior neuromuscular function supported by their relatively higher vitamin D levels. However, it should be noted that although their vitamin D levels are relatively higher within the group, they still fall within a low range compared to healthy elderly populations. This insufficiency may have already exerted a covert adverse effect on bone mass maintenance and bone microarchitectural homeostasis, which should not be overlooked. Consistent with our findings, Monica et al. noted that distal radius fractures in the elderly often present earlier than hip and vertebral fractures and frequently involve underlying abnormalities in bone mass and microstructure (5). For patients with multiple fractures, since those with concurrent hip fractures account for 89.94% of this group, the underlying influencing mechanisms are similar to those for hip fracture patients. Notably, given the cross-sectional design, causal inferences cannot be drawn from these observations. Although our study provides valuable insights into the relationship between serum vitamin D levels and fracture site specificity in older adults with fragility fractures, further research is needed to elucidate the underlying causal mechanisms.

Consistent with this site-specific pattern, previous randomized controlled trials have reported heterogeneous efficacy of vitamin D supplementation across fracture types. For instance, a study by Mitsutaka et al. found that vitamin D supplementation significantly reduced the incidence of hip fractures, whereas no statistically significant reduction was observed for vertebral or distal radius fractures (35). Similarly, a meta-analysis by Heike et al. demonstrated that oral vitamin D supplementation (700–800 IU/d) reduced the risk of hip and nonvertebral fractures in ambulatory or institutionalized older adults (36). Given the cross-sectional design, our findings cannot establish causality and should not be interpreted as explaining the results of intervention trials. Prospective studies are needed to determine whether baseline vitamin D status differentially influences fracture site susceptibility and whether site-specific supplementation strategies could optimize fracture prevention.

Existing studies have shown that serum 25(OH)D concentration is significantly associated with adverse outcomes following fractures (25, 26). Notably, in the present study, elderly patients with fragility fractures exhibited a high prevalence of vitamin D deficiency (35.94%), with a marked site-specific distribution pattern: the prevalence of deficiency increased stepwise from 24.35% in vertebral fractures to 44.38% in multiple fractures. Among all fracture types, patients with multiple fractures and hip fractures had both the highest deficiency rates and the lowest serum 25(OH)D concentrations. These findings underscore the need to prioritize these patient groups for vitamin D status assessment and targeted intervention. For patients with wrist or vertebral fractures, although vitamin D insufficiency remains common, screening may be assigned a lower priority compared to those with hip or multiple fractures. This risk-based stratification strategy may improve the efficiency and equity of healthcare resource allocation by prioritizing early identification and standardized management of high-risk patients. However, these recommendations are derived from cross-sectional observational data, which precludes causal inference. Their clinical utility therefore awaits confirmation in rigorously designed, prospective interventional trials.

The principal strengths of this study include its large-scale, phenotypically well-characterized cohort (*n* = 2,543), rigorous adjustment for key confounders (age, sex, blood sampling season, and comorbidities), and precise clinical stratification by fracture site. Nevertheless, several limitations warrant acknowledgment. First, as a cross-sectional design, the study cannot establish causal relationships, longitudinal data are required to enable robust causal inference. Second, the single-center design may limit the generalizability of the findings. Third, this study included only hospitalized patients and did not include those treated on an outpatient basis, which may introduce selection bias. Fourth, owing to incompleteness in the electronic medical record data, factors affecting serum 25(OH)D concentrations, including vitamin D or calcium supplementation, bone mineral density, body mass index (BMD), physical activity, institutionalization, smoking, alcohol use, and nutritional status, were not included in the multivariable analysis. These unmeasured covariates may introduce residual confounding, representing an important limitation of this study. For instance, given the well-recognized site-specific differences in bone mineral density, the observed site-specific variations in vitamin D levels may be partly attributable to underlying differences in baseline BMD across fracture types, rather than representing an independent effect of vitamin D. Similarly, differential vitamin D or calcium supplementation across fracture groups may influence serum 25(OH)D concentrations. Patient characteristics such as age and sex vary across groups, potentially affecting supplement awareness and adherence. Additionally, poorer functional status reduces physical activity, leading to less sun exposure and lower vitamin D synthesis, while also independently increasing fracture risk. Although we adjusted for some mobility-limiting comorbidities (e.g., cerebral infarction, COPD, and coronary artery disease), the lack of more direct data on functional status and physical activity may further confound the observed associations. Thus, future studies should include these potential confounders in their analyses. Despite these limitations, this study provides high-quality evidence on vitamin D nutritional status in elderly patients with fragility fractures, supported by its large sample size and standardized measurement of serum 25(OH)D concentrations, thereby establishing a scientific foundation for future clinical intervention studies and public health policy development.

## 5 Conclusion

In conclusion, vitamin D deficiency is highly prevalent among older adults with fragility fractures, and is significantly associated with advancing age, female sex, winter season, and history of cerebral infarction. Notably, serum 25(OH)D concentrations differed significantly across fracture sites, with the lowest levels observed in hip and multiple fractures and the highest in vertebral fractures. This difference may reflect the dual role of vitamin D in neuromuscular function and bone metabolism, underscoring the need for longitudinal interventional studies to further explore these relationships.

## Data Availability

The raw data supporting the conclusions of this article will be made available by the authors, without undue reservation.
