# Supplementary material for: Vitamin D status and site-specific fracture pattern associations in older adults with fragility fractures: a cross-sectional analysis of 2543 patients
Source: Front Nutr. 2026 Apr 24;13:1810545. doi: 10.3389/fnut.2026.1810545 (PMC13154272; doi:10.3389/fnut.2026.1810545)
Supplement: Supplementary file 1 [file Table_1.docx]

Supplementary Material

**Vitamin D Status and Site-Specific Fracture Pattern Associations in Older Adults with Fragility Fractures: A Cross-Sectional Analysis of 2543 Patients**


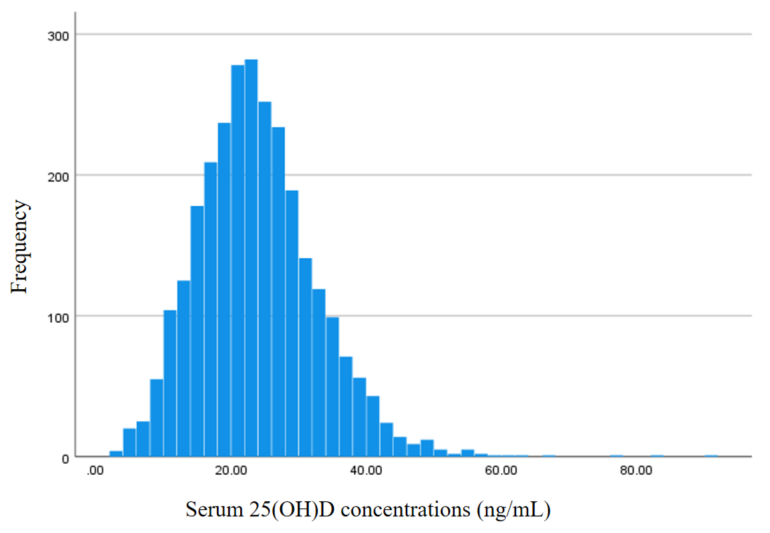


Supplementary Figure S1. Frequency distribution of vitamin D concentration.


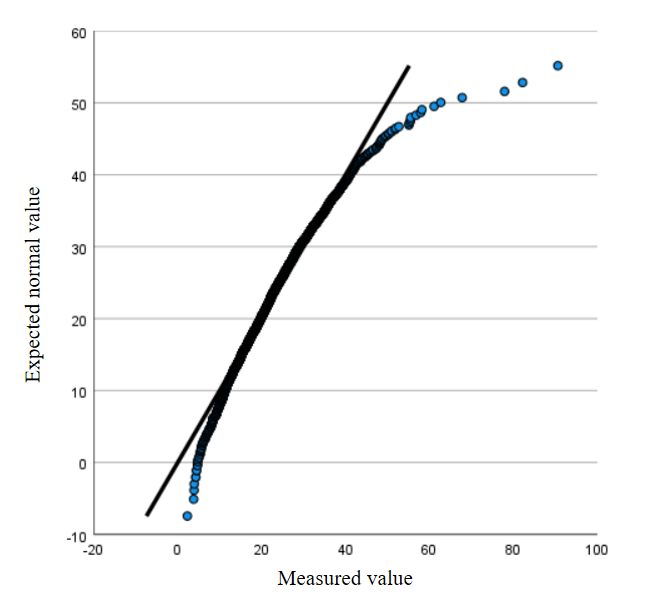


Supplementary Figure S2. Q-Q (quantile-quantile) plot assessing the normality of vitamin D level distribution. Departures of the data points (circles) from the diagonal reference line (solid) suggest deviations from a normal distribution.

Supplementary Table S1. Comparison of sersum 25(OH)D concentrations in four seasons.

| Season | Spring | Summer | Autumn |
| --- | --- | --- | --- |
| Summer | <0.001^✱^ |  |  |
| Autumn | 1.000 | 0.004^✱^ |  |
| Winter | 0.735 | <0.001^✱^ | 0.359 |

*Variables with *p*-value < 0.05.

Supplementary Table S2. Comparison of serum 25(OH)D concentrations in five age groups

| Age（years） | 60-64y | 65-69y | 70-74y | 75-79y | 80-84y |
| --- | --- | --- | --- | --- | --- |
| 65-69y | 1.000 |  |  |  |  |
| 70-74y | 0.122 | 0.370 |  |  |  |
| 75-79y | 0.016^✱^ | 0.049 | 1.000 |  |  |
| 80-84y | <0.001^✱^ | <0.001^✱^ | 0.014^✱^ | 0.196 |  |
| ≥85y | <0.001^✱^ | <0.001^✱^ | <0.001^✱^ | 0.001^✱^ | 1.000 |

*Variables with *p*-value < 0.05.

Supplementary Table S3. Comparison of serum 25(OH)D concentrations across four fracture sites.

| Fracture sites | Hip | Wrist | Vertebral |
| --- | --- | --- | --- |
| Wrist | 0.526 |  |  |
| Vertebral | < 0.001* | 0.064 |  |
| Multi-site | 0.275 | 0.229 | < 0.001* |

*Variables with *p*-value < 0.05.
